# A Modern Mode of Activation for Nucleic Acid Enzymes

**DOI:** 10.1371/journal.pone.0000673

**Published:** 2007-07-25

**Authors:** Dominique Lévesque, Francis P. Brière, Jean-Pierre Perreault

**Affiliations:** RNA Group/Groupe ARN, Département de Biochimie, Faculté de médecine et des sciences de la santé, Université de Sherbrooke, Sherbrooke, Québec, Canada; National Institute on Aging, United States of America

## Abstract

Through evolution, enzymes have developed subtle modes of activation in order to ensure the sufficiently high substrate specificity required by modern cellular metabolism. One of these modes is the use of a target-dependent module (i.e. a docking domain) such as those found in signalling kinases. Upon the binding of the target to a docking domain, the substrate is positioned within the catalytic site. The prodomain acts as a target-dependent module switching the kinase from an *off* state to an *on* state. As compared to the allosteric mode of activation, there is no need for the presence of a third partner. None of the ribozymes discovered to date have such a mode of activation, nor does any other known RNA. Starting from a specific on/off adaptor for the hepatitis delta virus ribozyme, that differs but has a mechanism reminiscent of this signalling kinase, we have adapted this mode of activation, using the techniques of molecular engineering, to both catalytic RNAs and DNAs exhibiting various activities. Specifically, we adapted three cleaving ribozymes (hepatitis delta virus, hammerhead and hairpin ribozymes), a cleaving 10-23 deoxyribozyme, a ligating hairpin ribozyme and an artificially selected capping ribozyme. In each case, there was a significant gain in terms of substrate specificity. Even if this mode of control is unreported for natural catalytic nucleic acids, its use needs not be limited to proteinous enzymes. We suggest that the complexity of the modern cellular metabolism might have been an important selective pressure in this evolutionary process.

## Introduction

**Figure 1:**
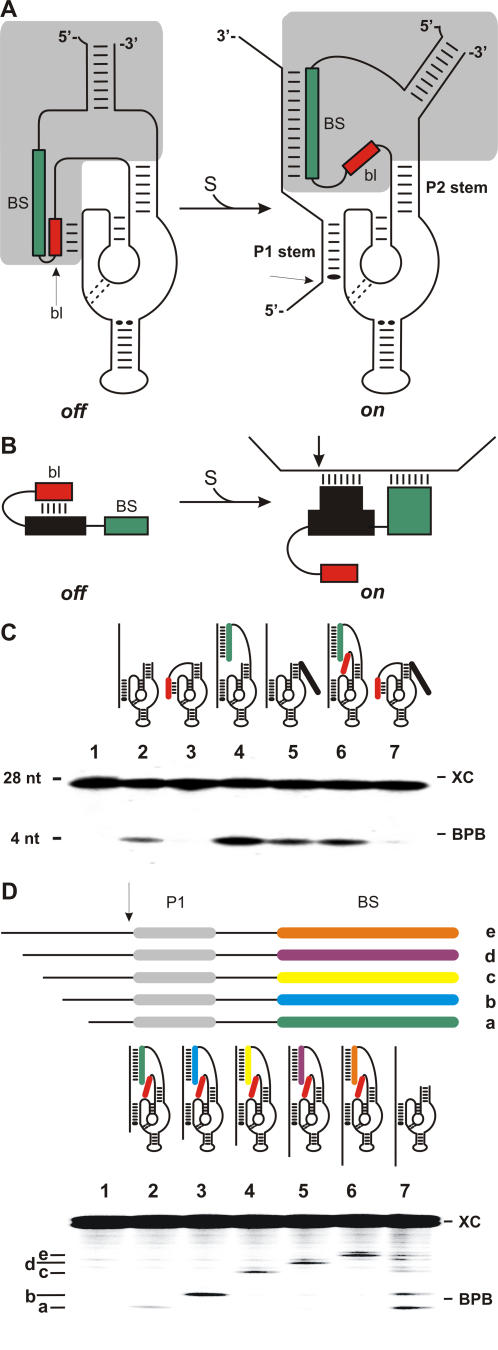
SOFA module on the HDV ribozyme. (A) Secondary structure of both the *off* and *on* conformation of the SOFA module (in grey) engineered on the P2 stem of the HDV ribozyme. The biosensor (BS, in green) and the blocker (bl, in red) are juxtaposed. (B) Schematic representation of separated blocker and biosensor domains modulating the activity of a ribozyme in a substrate-dependent manner. (C) Autoradiogram of cleavage assays performed with various HDV ribozymes. Their structures, with both the biosensor (green) and the blocker (red), are illustrated above the lanes of the gel. The control (-) was performed in the absence of ribozyme (lane 1), while lane 2 was performed in the presence of the original version. Lane 3 is the version extended by the blocker sequence only. Lanes 4 and 5 are the versions extended by a biosensor domain either complementary, or not, to the substrate, respectively. Finally, lanes 6 and 7 are the *on* and *off* versions including separated blocker and biosensor domains, respectively. BPB and XC indicate the positions of the bromophenol blue and xylene cyanol dyes, respectively. The nucleotide sequences of each of the engineered ribozyme are shown in the Figure S1. (D) Verification of the gain in substrate specificity of the *trans*-acting HDV ribozymes. The upper portion shows a schematic representation of the substrates in which the P1 binding domain is identical (grey), but the sequences of the biosensors vary (different colors). The lower portion shows the autoradiogram of a cleavage assay performed on a pool of substrates either by a specific HDV *on* ribozyme (a to e correspond to the cleavage produced by specific ribozymes, lanes 2 to 6, respectively), or by the original ribozyme (lane 7). Lane 1 is the negative control performed in the absence of ribozyme. The nucleotide sequences of each of ribozyme are shown in the Figure S2.

Enzymes have evolved various versions of a “molecular switch” with which they control their catalytic activities. For example, regulatory proteins bind effectors that induce conformational changes that control catalytic activity [1]. Similar modes of activation have been adopted by catalytic RNA motifs such as the *glm*S riboswitch, a ribozyme that regulates gene expression in a unique fashion through autocatalytic cleavage upon its binding to glucosamine-6-phosphate [2], [3]. Mitogen-activated protein kinases (MAP kinases) have evolved a similar, but more subtle, mode of activation [4], [5]. Upon the binding of the target to a docking domain, the substrate is positioned within the catalytic site. The prodomain acts as a target-dependent module switching the kinase from an *off* state to an *on* state, a property which considerably improves the substrate specificity of the enzyme. None of the ribozymes discovered to date exhibit such a mode of activation, nor does any other known RNA. However, a Specific *On*/*ofF* Adaptor (SOFA) module, which includes both a blocker and a biosensor domain, has been developed for the hepatitis delta virus (HDV) ribozyme [6], [7]. The rationale behind this design was that in the absence of the target, the ribozyme should be turned *off* by the addition of a blocker domain (bl) that forms an intramolecular stem with a portion of the substrate recognition domain (stem P1; Figure 1A). Conversely, a biosensor domain (BS) must bind its complementary sequence on the substrate in order to unlock the catalytic core (turning *on*). In the SOFA module the blocker and biosensor are juxtaposed, which is not the case for the domain involved in the activation of MAP kinases. In the present work, we have adapted this mode of activation, using the techniques of molecular biology, to a wide range of catalysis types exhibited by nucleic acids.

## Results

### Development of a new on/off adaptor version for HDV ribozyme

Initially, a new design that considers the blocker and biosensor domains as distinct entities was performed by including them at each end of the P2 stem of the HDV ribozyme in a process that involved several steps (Figure 1B). First, a blocker sequence that base-pairs with the binding domain of the ribozyme was added to the 5′-end. The resulting ribozyme exhibited no cleavage activity with a model substrate derived from the internal ribosome entry site (IRES) region of the hepatitis C virus (HCV) (Figure 1C, lane 3). The blocker acts as a “safety lock” placing the ribozyme in an inactive conformation (*off* state). When the ribozyme possesses a 12 nucleotide (nt) biosensor sequence complementary to the substrate at its 3′-end, it exhibited an improved cleavage activity (Figure 1C, lane 4). The presence of an inappropriate biosensor domain (i.e. a sequence not complementary to the substrate) resulted in a cleavage level comparable to that of the original ribozyme (Figure 1C, lane 5). When an appropriate biosensor was added to a ribozyme that already possessed a blocker, a dramatic increase in the cleavage activity, as compared to that observed in the presence of an inappropriate biosensor, was observed (Figure 1C, lanes 6 and 7, respectively). Thus, the ribozyme can be switched *off* and *on* by independent domains in a way reminiscent of the MAP kinases.

Kinetic analyses were performed in order to determine the second order rate constants (k_cat_/K_M_') as an indication of the substrate specificities. The HDV *on* (i.e. the one including both a blocker and an appropriate biosensor) exhibited a cleavage activity characterized by a relative k_cat_/K_M_' 6-fold higher than that of the original ribozyme, which in turn had a value 12-fold higher than that of the HDV *off* ribozyme (i.e the one including a blocker and an inappropriate biosensor) (see Table 1). The difference between the k_cat_/K_M_' values of the *off* and *on* ribozymes is therefore greater than 60 times. This difference is smaller than the determined 1500 to 2500 times observed between the *on* and *off* forms of the original configuration of the SOFA module on other HDV ribozymes [6], [7]. The larger difference in the latter case is likely due to the fact that its configuration is probably better adapted to the architecture of this particular ribozyme.

**Table 1:** Kinetic parameters of four cleaving nucleic acid enzymes in single turnover conditions

nucleic acid enzymes	k_cat _(min^−1^)	K_M' _(nM)	k_cat_/K_M' _(min^−1^/M^−1^)	relative k_cat_/K_M' _values
HDV wt	0.22±0.03	128±37	1.7×10^6^	12
HDV *on*	0.11±0.01	12±3	9.2×10^6^	66
HDV *off*	0.034±0.004	240±82	1.4×10^5^	1
hammerhead wt	0.074±0.002	48±8	1.5×10^6^	1765
hammerhead *on*	0.13±0.01	59±24	2.2×10^6^	2588
hammerhead *off*	6.38×10^−5^±0.70×10^−5^	75±28	8.5×10^2^	1
deoxyribozyme wt	0.76±0.08	211±45	3.6×10^6^	1714
deoxyribozyme *on*	1.80±0.12	312±38	5.8×10^6^	2762
deoxyribozyme *off*	1.24×10^−4^±0.04×10^−4^	60±7	2.1×10^3^	1
hairpin wt	0.41±0.02	163±22	2.5×10^6^	2083
hairpin *on*	0.12±0.01	11±2	1.1×10^7^	9167
hairpin *off*	2.8×10^−4^±0.6×10^−4^	240±155	1.2×10^3^	1

wt indicates wildtype

As the specificity of a ribozyme is commonly defined as its ability to discriminate between similar substrates [1], a collection of five substrates possessing identical ribozyme binding domains, but different biosensor sequences, was synthesized (Figure 1D). Because these substrates harbor different 5′-ends, they can be distinguished by their electrophoretic migration (Figure 1D, lane 1). When the 5 substrates were incubated together with only one HDV *on* ribozyme, solely the substrate possessing the sequence perfectly complementary to the biosensor was cleaved (Figure 1D, lanes 2-6). Conversely, an original ribozyme (i.e. one without both a biosensor and a blocker) had the ability to cleave all of the substrates, although at different levels (Figure 1D, lane 7). Together, these data demonstrate the improvement, in terms of substrate specificity, resulting from the addition of both a blocker and a biosensor. Moreover, it shows that a ribozyme, when activated by the proper substrate, does not cleave other substrates via a *trans*-cleavage mechanism. Finally, the potential to cleave HCV-derived transcripts was tested in cell culture (data not shown). Only small amounts of transcripts were cleaved. Determination of the half-life of the new conformation of the SOFA-HDV ribozymes according to a procedure reported previously [8], revealed a relatively low molecular stability of these ribozymes (a likely by-product of the single-stranded extremities that these ribozymes possess). More important is that it has been possible to adapt the target-dependent module of activation from the MAP kinase to the HDV ribozyme.

### Adaptation of this mode of activation to various cleaving nucleic acid enzymes

**Figure 2:**
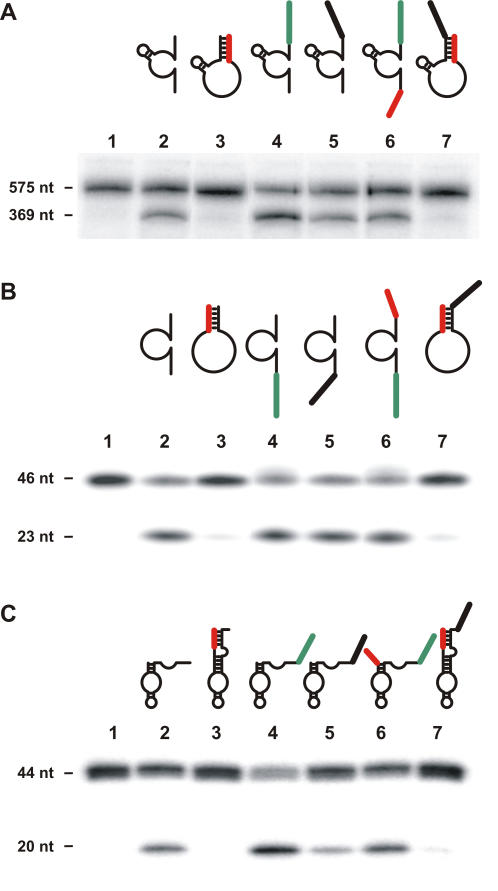
Autoradiograms of cleavage assays performed with controlled cleaving nucleic acid enzymes. (A) (B) and (C) are the reactions performed with the hammerhead ribozyme, the 10-23 deoxyribozyme and the hairpin ribozyme, respectively. In each case the structures of the nucleic acid enzyme with the blocker (red) and biosensor (green) are illustrated above the appropriate lanes of the gels, and the control (-) was performed in the absence of any nucleic acid enzyme (lane 1), and, lane 2, in the presence of the unmodified version of the ribozyme. Lane 3 is the version extended by the blocker sequence. Lanes 4 and 5 are the versions extended by a biosensor that is either complementary, or not, to the substrate. Finally, lanes 6 and 7 are the *on* and *off* versions, respectively. The nucleotide sequences of each nucleic acid enzyme are depicted in the Figures S3 [Supplementary-material pone.0000673.s004] to S5.

Subsequently, we tried to adapt this mode of activation to other catalytic nucleic acids. As a first attempt, we chose three cleaving molecules: the hammerhead ribozyme, the 10-23 deoxyribozyme and the hairpin ribozyme (Figure 2) [9]–[11]. All of these nucleic acid enzymes share a relatively similar architecture. The cleavage site of the substrates is located in the middle of the two stems formed between the catalytic center. The sequences of both of these ribozymes and of the deoxyribozyme have been reported to cleave various substrates that were used [12]–[15]. Each of these catalytic nucleic acids showed significant cleavage of their 5′-end labelled substrates, although at different levels (Figure 2A–C, lane 2). The addition of a blocker domain that “zips off” the binding domain dramatically decreased the cleavage activity (i.e. Figure 2A–C, lane 3). As is observed with the biosensor-blocker HDV ribozyme, the specificity of these engineered nucleic acid molecules was dramatically increased through blocker-mediated inactivation followed by reactivation via an extended complementarity with the appropriate biosensor sequence (Figure 2A–C, lanes 4 to 7). With both the biosensor and blocker domains, the presence of an inappropriate biosensor locked the catalytic core into an *off* state, while the presence of an appropriate biosensor permitted the switch into the active mode, by displacing the blocker, solely in presence of its specific substrate. The biosensors interact through more base pairs than do the blockers, thereby favoring the *on* state over the *off* state. When comparing the k_cat_/K_M'_ values for the *off* and *on* versions of these catalytic molecules, the overall improvements were of a minimum of three orders of magnitude (Table 1). Specifically, the k_cat_/K_M'_ values vary from 2588-, 2762- and 9167-folds between the *off* and *on* version of hammerhead ribozyme, hairpin ribozyme and 10-23 deoxyribozyme, respectively. This illustrates the important gain in terms of specificity. These important differences were mainly the result of dramatic decreases in the k_cat_ values between those of the original structures and those of their respective *off* states, suggesting that they lost their abilities to perform non-specific cleavage. Importantly, the addition of both a blocker and a biosensor permits the generation of cleaving nucleic acid enzymes that operate in a target-dependent fashion.

### Engineering ribozymes catalyzing various reactions

**Figure 3:**
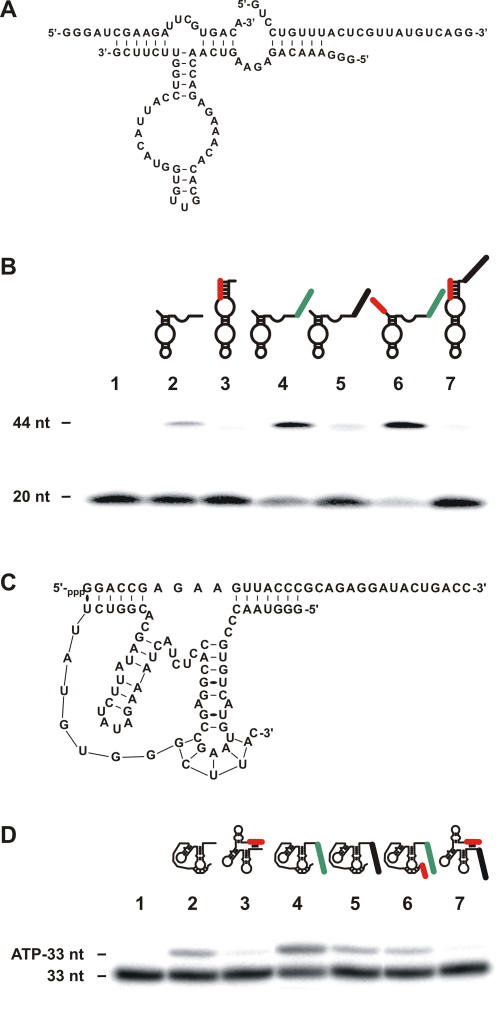
Catalytic activity of the engineered dependent ligating and capping ribozymes. (A) and (C) are the nucleotide sequences and secondary structures of the ribozyme-substrate complexes of the ligating hairpin and capping ribozymes, respectively. (B) and (D) are autoradiograms of the PAGE gels used to analyse the ligation and capping reactions, respectively. The schematic structures of the nucleic acid enzymes with both the blocker (red) and biosensor (green) are illustrated above the appropriate lanes of the gels. The controls (-) was performed in the absence of ribozyme (lane 1), and, lane 2, in the presence of the unmodified versions. Lane 3 is the ribozyme extended by the blocker sequence. Lanes 4 and 5 are the versions extended by a biosensor that is either complementary, or not, to the substrate, respectively. Finally, lanes 6 and 7 are the *on* and *off* ribozymes, respectively. The nucleotide sequences of each engineered nucleic acid enzyme are depicted in Figures S6 and S7.

Next, we investigated other ribozyme's catalyses. As a first attempt, the ligation catalyzed by the hairpin ribozyme was studied. Extending the 3′-end of the original hairpin ribozyme by 6 nt resulted in the retention of the cleavage product, and consequently favored the reverse reaction (Figure 3A) [14]. Using a 5′-^32^P-labelled RNA strand possessing a terminal 2′-3′-cyclic phosphate and a second RNA strand possessing a 5′-hydroxyl resulted in the detection of ligation products (Figure 3B). The addition of a biosensor with a sequence complementary to that of the substrate increased the amount of ligation observed. In contrast, the addition of the blocker module almost completely abolished the ligation. Finally, an *on* version, which includes both the blocker and an appropriate biosensor, exhibited a ligation activity of up to 65%; while an *off* version (i.e. one with an inappropriate biosensor) showed less than 5% activity.

Subsequently, the concept was adapted to an artificial ribozyme that catalyzed the attack of a 5′-terminal nucleotide phosphate on the α-phosphate of the terminal triphosphate of an RNA substrate, forming a 5′-5′ phosphodiester linkage (i.e. a capping ribozyme; Figure 3C–D) [16]. A version harboring a blocker alone exhibited only residual activity for the capping of a 33-nucleotide substrate (<3%), while one possessing only an appropriate biosensor sequence was slightly more efficient than the original. The *on* version has the ability to switch from its *off* to *on* state, while the *off* version remained trapped in the inactive conformation.

## Discussion

Together, these experiments demonstrate the possibility of adapting the target-dependent activation concept to various types of nucleic acid enzymes. The limiting factor with such an adaptation is the fact that the suitable candidates need to have a recognition mechanism based on base pair formation in order to permit a rational design. As compared to the allosteric mode of activation that has been engineered for synthetic nucleic acid circuits based on oligonucleotide-sensing allosteric ribozymes and deoxyribozyme [17]–[19], in this case there is no need for the presence of a third partner.

As first glance it might appear somewhat surprising that any known natural catalytic RNA would resort to this mode of regulation in order to provide the appropriate substrate specificity. This might be a mode of regulation for processes that require additional selective pressures in order to ensure a greater fidelity of action, for example as it is required for the MAP kinases. The complexity of the modern cellular metabolism might have been an important selective pressure in this evolutionary process. Catalytic RNAs might not have needed to evolve such a regulation mechanism due to the fact that their recognition strategy, which is based on base pair formation, provided sufficient specificity. Moreover, these ribozymes act in *cis*, therefore there is no requirement for such mechanism. Other natural ribozymes, such as the ribonuclease P [20], ribosome [21] and spliceosome [22], catalyze their reactions with many substrates rendering high substrate specificity detrimental. Alternatively, the ability of *cis*-acting ribozymes to adopt different conformations may have contributed to their regulation. For example, viroids self-cleave their nascent transcripts through a hammerhead structure, but when the polymerase progresses further, a sequence partially complementary to the self-cleavage motif is synthesized, and, consequently, a rod-like structure is folded [23]. In this structure, the hammerhead motif is in an *off* state that prevents cleavage after viroid circularization. Thus, examples of RNA evolution that have already reached the blocker step of this mode of activation do indeed exist. Here, we show that a target-dependent activation, analogous to that of the MAP kinases, is possible for a wide range of nucleic acid catalysis types. It is therefore tempting to speculate that it already exists in nature, and that it simply remains to be found.

## Materials and Methods

### DNA templates and constructs

Deoxyribozymes were purchased as oligonucleotides from Invitrogen. All ribozymes and small RNA substrates used were produced by PCR using oligonucleotides purchased from either Invitrogen or IDT Inc. Briefly, DNA oligonucleotides corresponding to the sequences complementary to the different templates, and possessing a T7 promoter complementary sequence, were annealed to T7 promoter primer and the primers extended in PCR reactions. The reaction products were then used as templates for run-off transcription (see below). Alternatively, some constructs were prepared by annealing of two overlapping oligonucleotides that were then filled in by PCR using Vent DNA polymerase (New England Biolab), producing a double-stranded DNA fragment. Each of these fragments was provided with a T7 promoter at the beginning of the sense strand for subsequent use as a template for run-off transcription. Hepatitis C virus (HCV) derived substrate (a 575 nt long template containing the internal ribosome entry site (IRES) and the first part of the ORF) was produced by *Kpn*I digestion of the pHCVA plasmid [6], which contains 1348 nt of the 5′ sequence of hepatitis C virus (genotype 1b) [24]. PCR products and linearized plasmid templates were purified by phenol/chloroform extraction, precipitated with ethanol and dissolved in water.

### RNA synthesis

Run-off transcriptions were performed as described previously [6]. Briefly, transcriptions were performed using purified T7 RNA polymerase (10 µg) in the presence of RNA Guard (24 U, Amersham Biosciences) and pyrophosphatase (0.01 U, Roche Diagnostics) in a buffer containing 80 mM HEPES-KOH, pH 7.5, 24 mM MgCl_2_, 2 mM spermidine, 40 mM DTT and either linearized plasmid DNA (5 µg) or PCR product (100 pmol) as template. Each NTP (5 mM) was added to the mixture either with or without 50 µCi of [α-^32^P]UTP (3 000 Ci/mmol, New England Nuclear) in a final volume of 100 µL, and the reaction incubated at 37°C for 2 to 4 h. Upon completion, the reaction mixtures were treated with DNase RQ1 (Promega) at 37°C for 20 min, and the RNA then purified by phenol:chloroform extraction and precipitation with ethanol. RNA products were fractionated by a denaturing (7 M urea) 6 to 20% polyacrylamide gel electrophoresis (PAGE; 19∶1 ratio of acrylamide to bisacrylamide) using 45 mM Tris-borate, pH 7.5/1 mM EDTA solution as running buffer. The reaction products were visualized either by UV shadowing, or by autoradiography. The bands corresponding to the correct sizes for both the ribozymes and the substrates were excised from the gel and the transcripts eluted overnight at room temperature in a buffer containing 1 mM EDTA, 0.1% SDS and 0.5 M ammonium acetate. The transcripts were ethanol precipitated at −80°C, washed, dried and dissolved in water. The amounts retrieved were determined either by ^32^P counting, or by absorbance at 260 nm.

In order to produce 5′-labelled RNA substrates, transcripts were dephosphorylated by adding 1 U of antartic phosphatase (New England Biolab) to 20 pmol of RNA and incubating for 30 min at 37°C in a final volume of 10 µL containing 50 mM Tris-HCl, pH 8.5, 0.1 mM EDTA and 40 U of RNAGuard (Amersham Biosciences). The enzyme was inactivated by incubating at 65°C for 5 min. Dephosphorylated RNA (5 pmol) was then radiolabelled at its 5′-end using 3 U of T4 polynucleotide kinase in the presence of 3.2 pmol of [γ-^32^P]ATP (6000 Ci/mmol, New England Nuclear). Radiolabelled transcripts were purified by electrophoresis on a denaturing gel and recovered as described above.

### Cleavage assays and kinetics

All reactions with the cleaving HDV, hammerhead and hairpin ribozymes, as well as those with the deoxyribozyme, were performed under single-turnover conditions ([Rz]>>[S]) using 100 nM of nucleic acid enzyme and trace amounts of 5′-^32^P-labelled substrates at 37°C in a final volume of 10 µL containing 50 mM Tris-HCl, pH 7.5 and 10 mM MgCl_2_. After an incubation of 1 h, the reactions were quenched by the addition of ice-cold formamide dye buffer (1 vol of 97% formamide, 10 mM EDTA, pH 8.0, 0.25% xylene cyanol, 0.25% bromophenol blue) and fractionated on 6% to 20% PAGE gels. The gels were then analyzed with a radioanalytic scanner (Storm, Molecular Dynamics). For time-course experiments, aliquots of 0.8 µL were taken at various times up to 3 h and treated as described above. Kinetic analyses were performed under single turnover conditions. Trace amounts of 5′-^32^P-labelled substrate (<1 nM) were cleaved by various concentrations of either ribozyme or deoxyribozyme (5–400 nM). The fraction cleaved was determined, and the rate of cleavage (k_obs_) obtained from fitting the data to the equation A_t_ = A_□_(1-e^−kt^) where A_t_ is the percentage of cleavage at time t, A_□_ is the maximum percent cleavage (or the end-point of cleavage), and k is the rate constant (k_obs_). Each rate constant was calculated from at least two independent measurements. The values of k_obs_ obtained were then plotted as a function of nucleic acid enzyme concentration for the determination of k_cat_, K_M_' and k_cat_/K_M_'. The values obtained from independent experiments varied by less than 15%. The experiments of substrate specificity using several substrates were performed under multiple turnover conditions using 100 nM HDV ribozyme and 1 µM substrate.

### Ligation reactions

Ligation reactions were performed as described previously [14], [15] with minor modifications. Full length 5′-^32^P-labelled substrate (5 pmol) was cleaved in the presence of 10 µM of hairpin ribozyme in a volume of 50 µl containing 50 mM Tris-HCl, pH 7.5 and 10 mM MgCl_2_. The mixture was incubated 2 h at 37°C, quenched by the addition of ice-cold formamide dye buffer, purified on a 20% denaturing PAGE gel and the radiolabelled 20 nt 5′-product recovered as described. This RNA strand was then used as substrate for the ligation reaction. The 3′-product used in this ligation reaction was purchased from IDT Inc. This deprotected 5′-hydroxyl RNA was purified as described above. Ligation reactions were performed using 10 µM of the hairpin ribozyme in the presence of 15 µM of the 3′-end substrate and trace amounts of 5′-^32^P-labelled substrate mixed in a total volume of 9 µl containing 50 mM HEPES-KOH, pH 7.0. The reactions were preheated at 37°C for 10 min so as to permit the annealing of the substrate to the ribozyme, and the ligation reactions started (time = 0) by adding 1 µL of 150 mM MgCl_2_. Samples were incubated at 37°C for 1 h, fractionated on gels and visualized by autoradiography.

### Capping enzyme reaction

The capping reaction was performed as described previously [16]. Substrate was 3′-end labelled by incubating, at 37°C, 10 pmol of transcripts with 3.2 pmoles of [^32^P]Cp (3000 ci/mmol, New England Nuclear) and 1 U T4 RNA ligase (New England Biolab) in the buffer supplied by the manufacturer supplemented with 10% DMSO. After 3 h, the reaction was stopped and the transcripts purified by denaturing 10% denaturing PAGE and recovered as described above. Capping reactions were carried out under single turnover conditions. Both the ribozyme (2 µM) and substrate (0.2 µM) were annealed together for 5 min at 22°C in a reaction buffer containing 50 mM Tris-HCl, pH 7.5, 25 mM MgCl_2_ and 150 mM KCl. The reactions were initiated by the addition of ATP to a final concentration of 1 mM. After 16 h of incubation at 22°C, the reactions were quenched by addition of ice-cold formamide dye buffer and analyzed on denaturing 15% PAGE gels.

## Supporting Information

Figure S1 Nucleotide sequence and secondary structure of the various versions of the HDV ribozyme cleaving a 28 nt substrate derived from the hepatitis C virus. (A) The original version. (B) The version including a 4 nt blocker (bl). (C) The version with an appropriate 12 nt biosensor (BS). (D) The version with an inappropriate 12 nt biosensor (iBS). (E) The on version with both a blocker and an appropriate biosensor. (F) The off version with both a blocker and an inappropriate biosensor (i.e. a biosensor of sequence not complementary to the substrate). The blocker, appropriate biosensor and inappropriate biosensor are in red, green and grey, respectively. The arrows indicate the cleavage sites.
(1.37 MB TIF)

Figure S2 Nucleotide sequence and secondary structures of the HDV ribozyme-substrate complexes for the substrate specificity experiment. All of the ribozymes and substrates possess the same P1 binding domain. (A) The original version of the HDV ribozyme cleaving a 28 nt substrate derived from the hepatitis C virus. (B) to (F) The HDV on ribozymes with the different biosensor modules (in different colors). (G) The original ribozyme that can bind all of the substrates, regardless of their binding domains to the biosensor.
(1.60 MB TIF)

Figure S3 Nucleotide sequence and secondary structure of the hammerhead ribozyme cleaving a 575 nt substrate derived from the HCV. (A) The original version as reported previously (12). (B) The version including an 8 nt blocker (bl). (C) The version with an appropriate 15 nt biosensor (BS). (D) The version with an inappropriate biosensor (iBS). (E) The on version with both a blocker and appropriate biosensor. (F) The off version with both a blocker and an inappropriate biosensor. The blocker, appropriate biosensor and inappropriate biosensor are in red, green and grey, respectively. The arrows indicate the cleavage sites.
(1.59 MB TIF)

Figure S4 Nucleotide sequence and secondary structure of the 10-23 deoxyribozyme cleaving a 46 nt substrate derived from the 5′ UTR of the human rhinovirus 14. (A) The original version as reported previously (13). (B) The version including an 8 nt blocker (bl). (C) The version with an appropriate 14 nt biosensor (BS). (D) The version with an inappropriate biosensor (iBS). (E) The on version with both a blocker and an appropriate biosensor. (F) The off version with both a blocker and an inappropriate biosensor. The blocker, appropriate biosensor and inappropriate biosensor are in red, green and grey, respectively. The arrows indicate the cleavage sites.
(1.18 MB TIF)

Figure S5 Nucleotide sequence and secondary structure of the hairpin ribozyme cleaving a 44 nt substrate. (A) The original version as reported previously (14). (B) The version including an 8 nt blocker (bl). (C) The version with an appropriate 11 nt biosensor (BS). (D) The version with an inappropriate biosensor (iBS). (E) The on version with both a blocker and appropriate biosensor. (F) The off version with both a blocker and an inappropriate biosensor. The blocker, appropriate biosensor and inappropriate biosensor are in red, green and grey, respectively. The arrows indicate the cleavage sites.
(1.69 MB TIF)

Figure S6 Nucleotide sequence and secondary structure of the hairpin ribozyme ligating two RNA strands 20 and 24 nt in length. (A) The original version. (B) The version including a 6 nt blocker (bl). (C) The version with an appropriate 11 nt biosensor (BS). (D) The version with an inappropriate biosensor (iBS). (E) The on version with both a blocker and appropriate biosensor. (F) The off version with both a blocker and an inappropriate biosensor. The blocker, appropriate biosensor and inappropriate biosensor are in red, green and grey, respectively.
(1.73 MB TIF)

Figure S7 Nucleotide sequence and secondary structure of the ribozyme capping a 33 nt RNA strand by an ATP molecule as reported previously (15). (A) The original version. (B) The version including a 5 nt blocker (bl). (C) The version with an appropriate 10 nt biosensor (BS). (D) The version with an inappropriate biosensor (iBS). (E) The on version with both a blocker and an appropriate biosensor. (F) The off version with both a blocker and an inappropriate biosensor. The blocker, appropriate biosensor and inappropriate biosensor are in red, green and grey, respectively.
(1.93 MB TIF)
