# Supplementary figures and images for: A Modern Mode of Activation for Nucleic Acid Enzymes
Source: PLoS One. 2007 Jul 25;2(7):e673. doi: 10.1371/journal.pone.0000673 (PMC1919428; doi:10.1371/journal.pone.0000673)

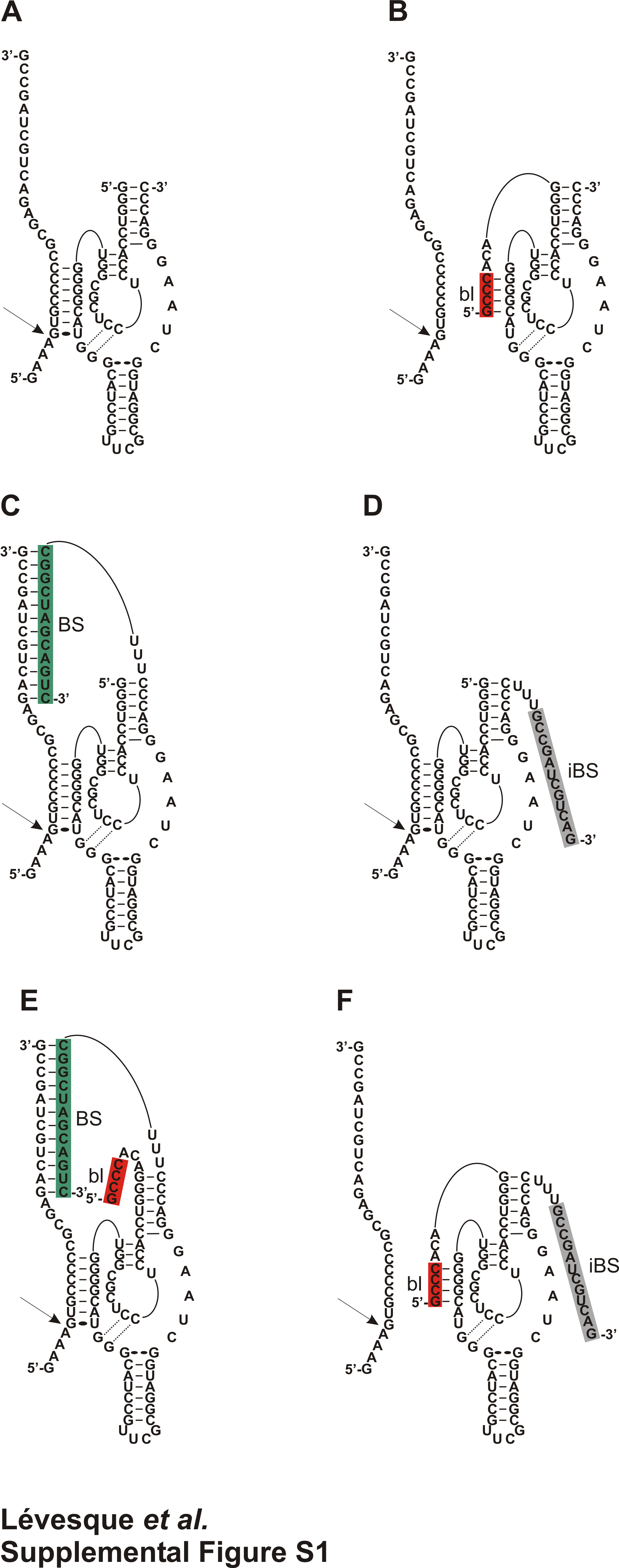

Supplement: Figure S1 — Nucleotide sequence and secondary structure of the various versions of the HDV ribozyme cleaving a 28 nt substrate derived from the hepatitis C virus. (A) The original version. (B) The version including a 4 nt blocker (bl). (C) The version with an appropriate 12 nt biosensor (BS). (D) The version with an inappropriate 12 nt biosensor (iBS). (E) The on version with both a blocker and an appropriate biosensor. (F) The off version with both a blocker and an inappropriate biosensor (i.e. a biosensor of sequence not complementary to the substrate). The blocker, appropriate biosensor and inappropriate biosensor are in red, green and grey, respectively. The arrows indicate the cleavage sites. (1.37 MB TIF) [file pone.0000673.s001.tif]

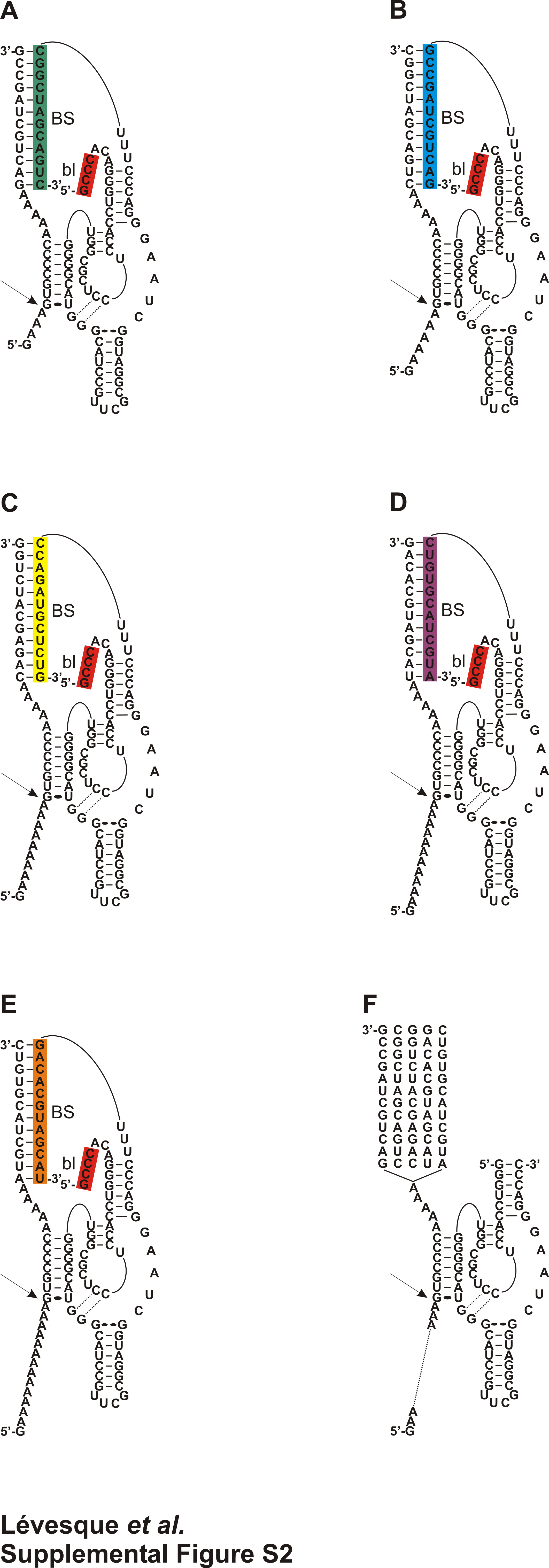

Supplement: Figure S2 — Nucleotide sequence and secondary structures of the HDV ribozyme-substrate complexes for the substrate specificity experiment. All of the ribozymes and substrates possess the same P1 binding domain. (A) The original version of the HDV ribozyme cleaving a 28 nt substrate derived from the hepatitis C virus. (B) to (F) The HDV on ribozymes with the different biosensor modules (in different colors). (G) The original ribozyme that can bind all of the substrates, regardless of their binding domains to the biosensor. (1.60 MB TIF) [file pone.0000673.s002.tif]

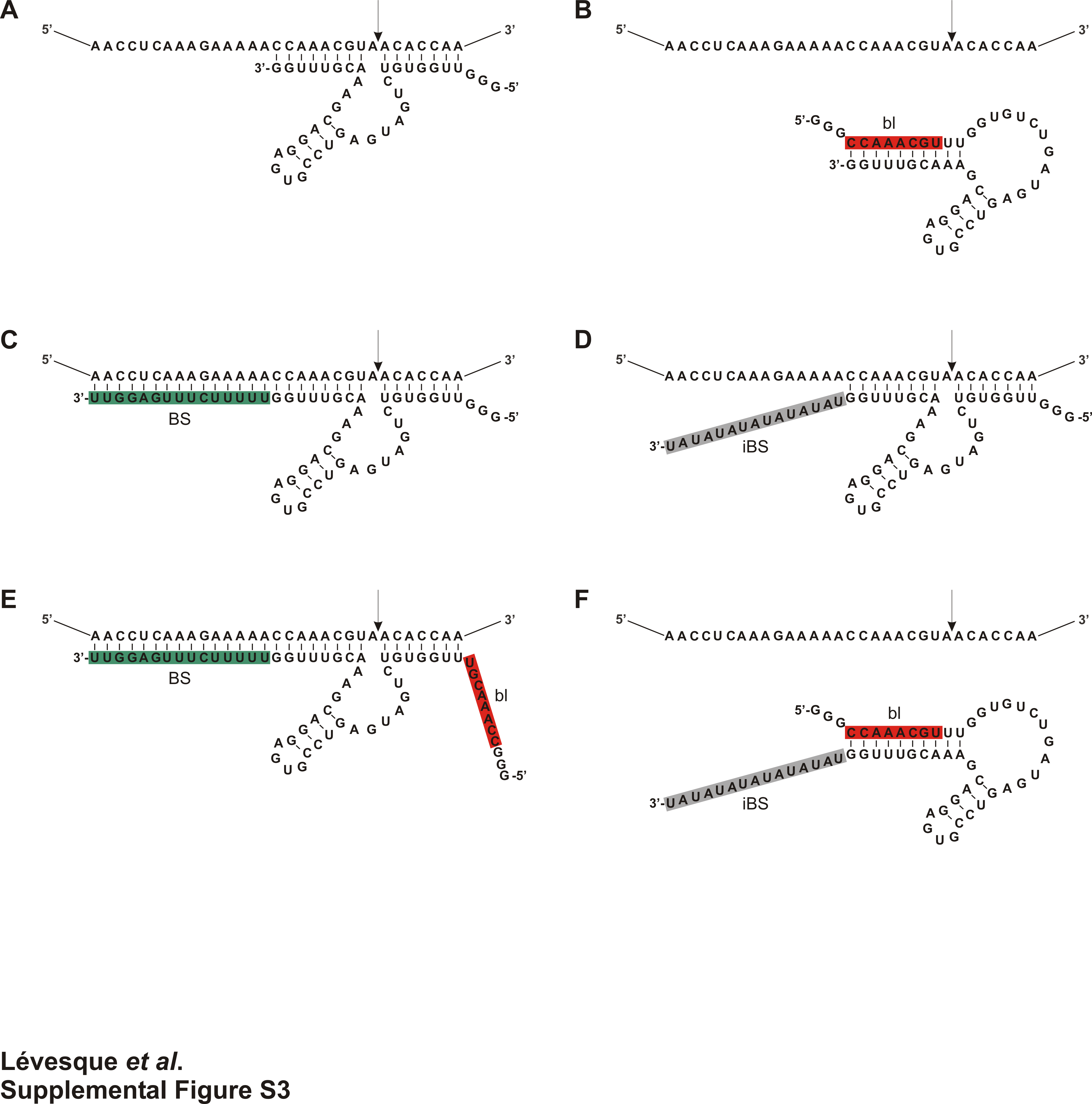

Supplement: Figure S3 — Nucleotide sequence and secondary structure of the hammerhead ribozyme cleaving a 575 nt substrate derived from the HCV. (A) The original version as reported previously (12). (B) The version including an 8 nt blocker (bl). (C) The version with an appropriate 15 nt biosensor (BS). (D) The version with an inappropriate biosensor (iBS). (E) The on version with both a blocker and appropriate biosensor. (F) The off version with both a blocker and an inappropriate biosensor. The blocker, appropriate biosensor and inappropriate biosensor are in red, green and grey, respectively. The arrows indicate the cleavage sites. (1.59 MB TIF) [file pone.0000673.s003.tif]

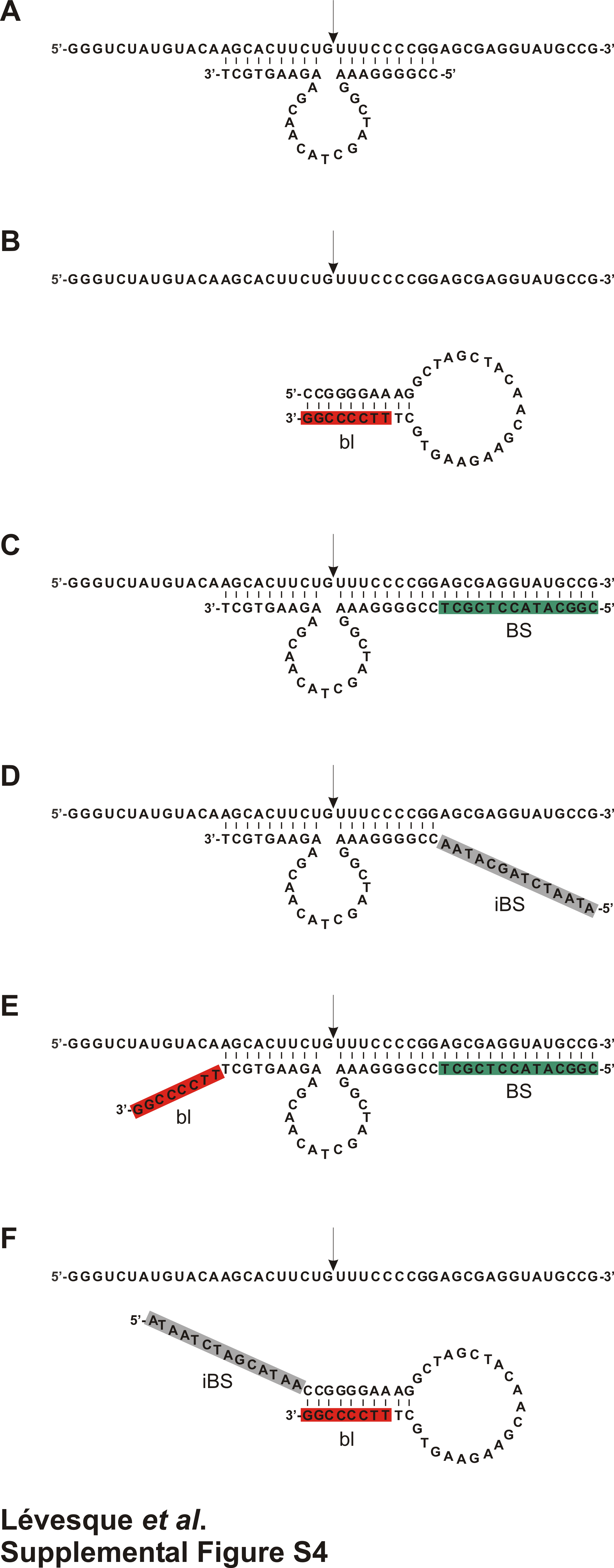

Supplement: Figure S4 — Nucleotide sequence and secondary structure of the 10-23 deoxyribozyme cleaving a 46 nt substrate derived from the 5′ UTR of the human rhinovirus 14. (A) The original version as reported previously (13). (B) The version including an 8 nt blocker (bl). (C) The version with an appropriate 14 nt biosensor (BS). (D) The version with an inappropriate biosensor (iBS). (E) The on version with both a blocker and an appropriate biosensor. (F) The off version with both a blocker and an inappropriate biosensor. The blocker, appropriate biosensor and inappropriate biosensor are in red, green and grey, respectively. The arrows indicate the cleavage sites. (1.18 MB TIF) [file pone.0000673.s004.tif]

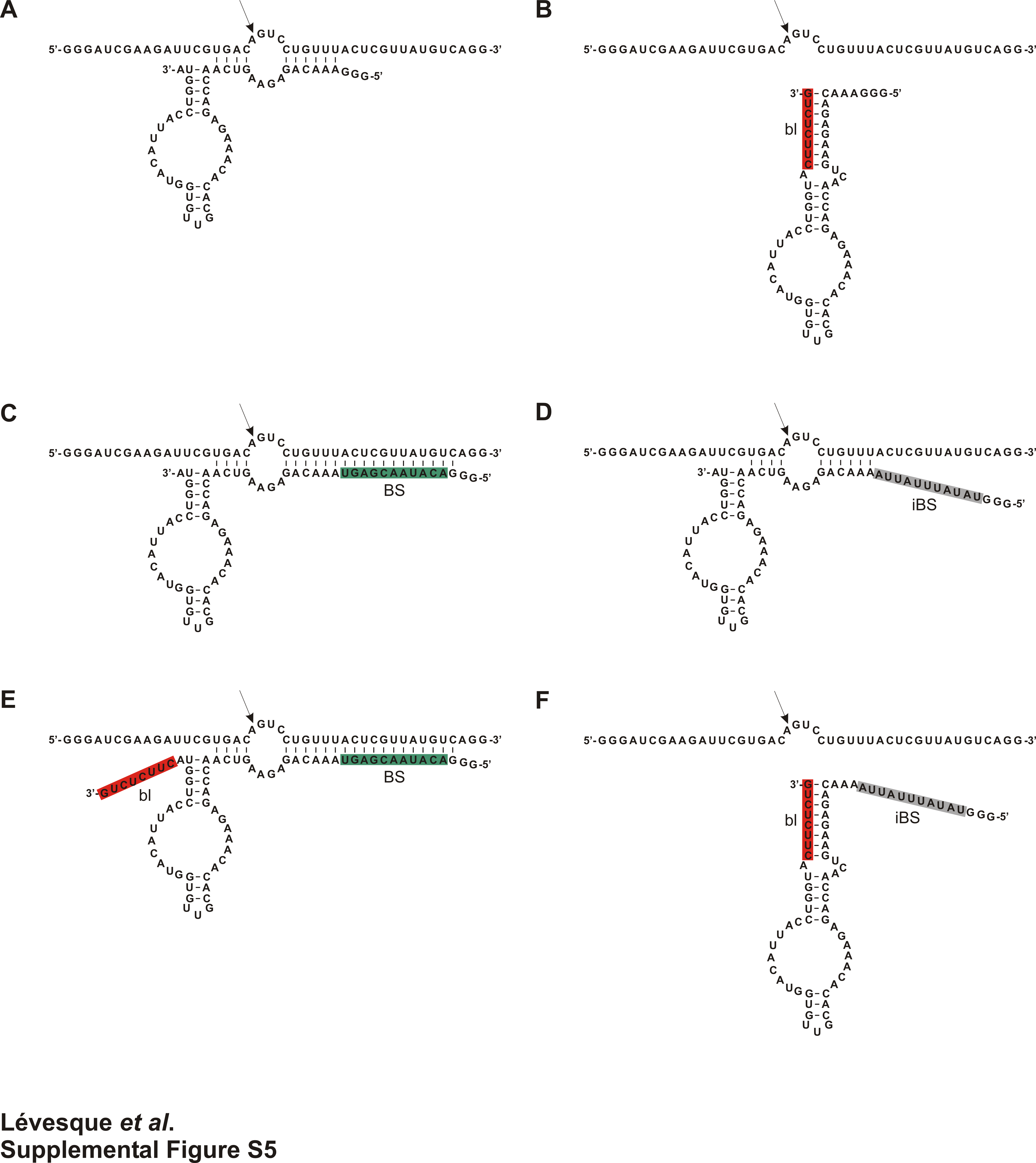

Supplement: Figure S5 — Nucleotide sequence and secondary structure of the hairpin ribozyme cleaving a 44 nt substrate. (A) The original version as reported previously (14). (B) The version including an 8 nt blocker (bl). (C) The version with an appropriate 11 nt biosensor (BS). (D) The version with an inappropriate biosensor (iBS). (E) The on version with both a blocker and appropriate biosensor. (F) The off version with both a blocker and an inappropriate biosensor. The blocker, appropriate biosensor and inappropriate biosensor are in red, green and grey, respectively. The arrows indicate the cleavage sites. (1.69 MB TIF) [file pone.0000673.s005.tif]

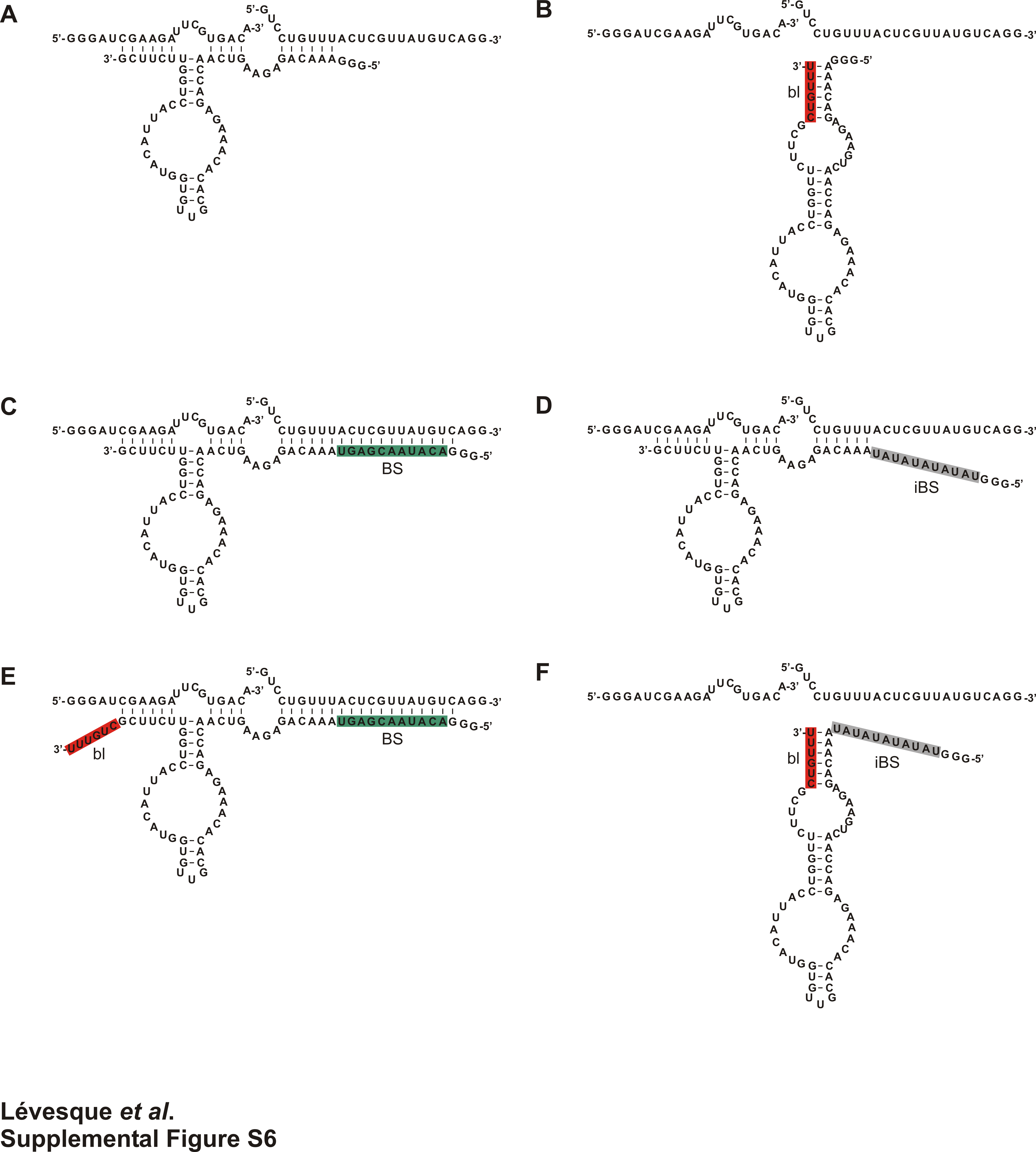

Supplement: Figure S6 — Nucleotide sequence and secondary structure of the hairpin ribozyme ligating two RNA strands 20 and 24 nt in length. (A) The original version. (B) The version including a 6 nt blocker (bl). (C) The version with an appropriate 11 nt biosensor (BS). (D) The version with an inappropriate biosensor (iBS). (E) The on version with both a blocker and appropriate biosensor. (F) The off version with both a blocker and an inappropriate biosensor. The blocker, appropriate biosensor and inappropriate biosensor are in red, green and grey, respectively. (1.73 MB TIF) [file pone.0000673.s006.tif]

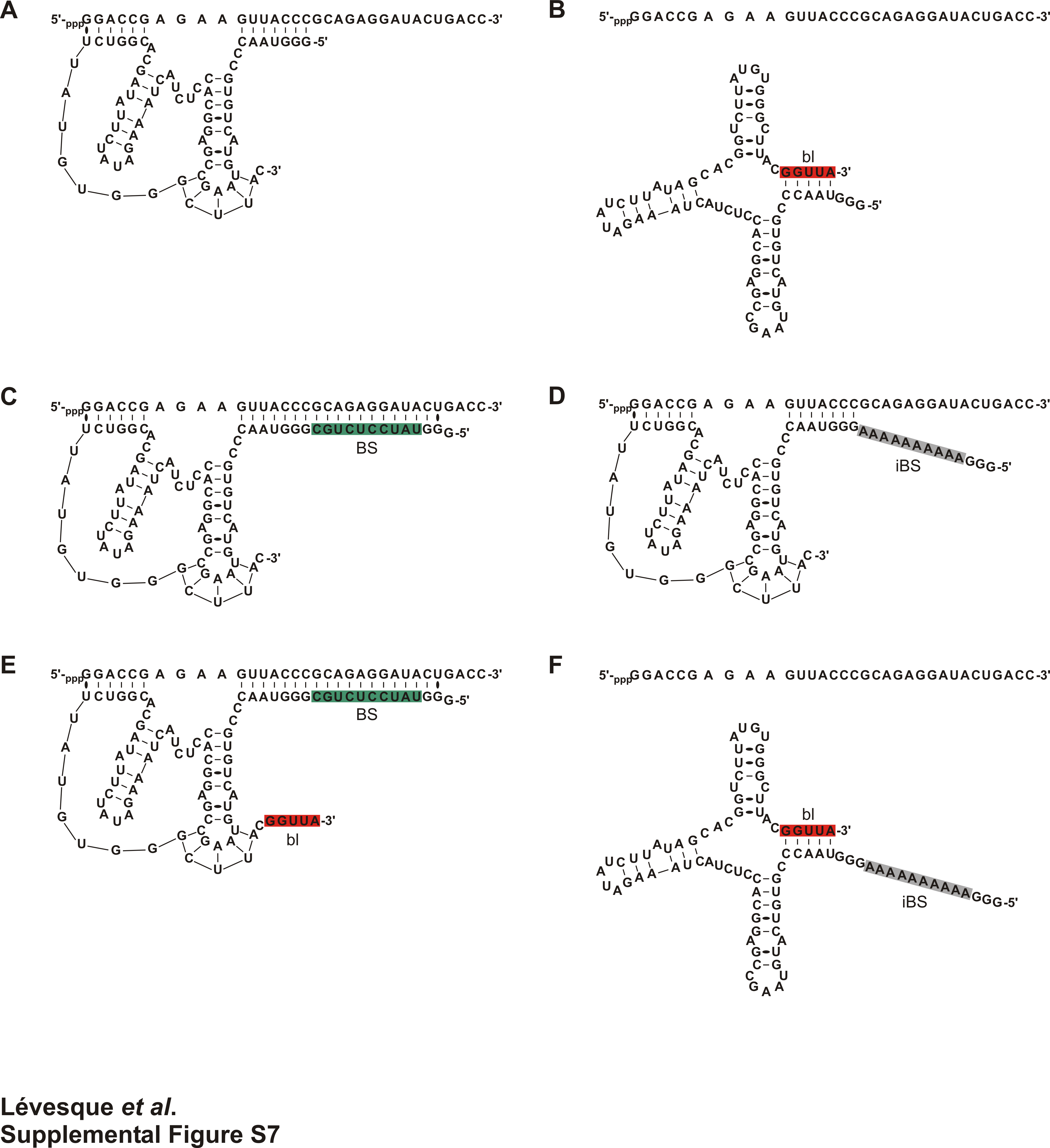

Supplement: Figure S7 — Nucleotide sequence and secondary structure of the ribozyme capping a 33 nt RNA strand by an ATP molecule as reported previously (15). (A) The original version. (B) The version including a 5 nt blocker (bl). (C) The version with an appropriate 10 nt biosensor (BS). (D) The version with an inappropriate biosensor (iBS). (E) The on version with both a blocker and an appropriate biosensor. (F) The off version with both a blocker and an inappropriate biosensor. The blocker, appropriate biosensor and inappropriate biosensor are in red, green and grey, respectively. (1.93 MB TIF) [file pone.0000673.s007.tif]
